# Detection of *KRAS* mutation using plasma samples in non-small-cell lung cancer: a systematic review and meta-analysis

**DOI:** 10.3389/fonc.2023.1207892

**Published:** 2023-07-06

**Authors:** Peiling Cai, Bofan Yang, Jiahui Zhao, Peng Ye, Dongmei Yang

**Affiliations:** ^1^ Department of Anatomy and Histology, School of Preclinical Medicine, Chengdu University, Chengdu, China; ^2^ School of Clinical Medicine, Chengdu University, Chengdu, China; ^3^ Clinical Laboratory & Clinical Research and Translational Center, Second People’s Hospital of Yibin City-West China Yibin Hospital, Sichuan University, Yibin, China

**Keywords:** KRAS, plasma, non-small cell lung cancer, diagnostic accuracy, meta-analysis

## Abstract

**Background:**

The aim of this study was to investigate the diagnostic accuracy of KRAS mutation detection using plasma sample of patients with non-small cell lung cancer (NSCLC).

**Methods:**

Databases of Pubmed, Embase, Cochrane Library, and Web of Science were searched for studies detecting KRAS mutation in paired tissue and plasma samples of patients with NSCLC. Data were extracted from each eligible study and analyzed using MetaDiSc and STATA.

**Results:**

After database searching and screening of the studies with pre-defined criteria, 43 eligible studies were identified and relevant data were extracted. After pooling the accuracy data from 3341 patients, the pooled sensitivity, specificity and diagnostic odds ratio were 71%, 94%, and 59.28, respectively. Area under curve of summary receiver operating characteristic curve was 0.8883. Subgroup analysis revealed that next-generation sequencing outperformed PCR-based techniques in detecting *KRAS* mutation using plasma sample of patients with NSCLC, with sensitivity, specificity, and diagnostic odds ratio of 73%, 94%, and 82.60, respectively.

**Conclusion:**

Compared to paired tumor tissue sample, plasma sample showed overall good performance in detecting KRAS mutation in patients with NSCLC, which could serve as good surrogate when tissue samples are not available.

## 1 Introduction

Lung cancer is a leading cause of cancer-related death worldwide (1). As its most prevalent subtype, non-small cell lung cancer (NSCLC) represents approximately 85% of lung cancer cases (2). Treatments of NSCLC include surgery, radiotherapy, chemotherapy, immunotherapy, and targeted therapy in tumors harboring certain oncogenetic variations, e.g., anti-epidermal growth factor receptor (EGFR) therapy (2).

*Kirsten rat sarcoma viral oncogene homologue* (*KRAS*) is the most frequently mutated oncogene in many types of cancer (3), with an overall prevalence of 27.5% in NSCLC (4). Mutation of *KRAS* gene is associated with resistance to anti-EGFR therapies (5–7). In addition, although KRAS was thought to be an “undruggable” target, it has become “druggable” after the successful approval of KRAS (G12C) inhibitor (Sotorasib) for the treatment of *KRAS* G12C-mutated metastatic NSCLC (8). Due to these important roles of *KRAS* mutation in targeted therapies, accurate detection of *KRAS* gene mutations, especially G12C, is crucial for the success of anti-EGFR therapies and KRAS inhibitors.

The detection of *KRAS* mutations in tumors is usually performed using tumor tissue samples, e.g., formalin-fixed paraffin-embedded (FFPE) tumor tissue samples. However, tissue samples are sometimes not available, or may not reflect the real-time mutation status of tumor due to the existence of cancer evolution (9). Research efforts were therefore made to find possible surrogates for tumor tissue samples, which are mainly cell-free DNA (cfDNA)-containing samples, such as plasma, urine, saliva, feces, exhaled breath condensate, and etc (10, 11). Before their clinical application, however, those surrogate sample types needs to be validated for their accuracy performance in detecting *KRAS* mutations. Many such studies have been conducted. A recently-published systemic review and meta-analysis by Palmieri (12) summarized the results of 40 relevant studies and reported an overall adequate accuracy of cfDNA-containing samples. This meta-analysis by Palmieri focused on cfDNA, and involved studies using plasma, urine, or sputum samples. However, cfDNA levels in the three sample types are quite different, which could potentially influence accuracy performance. In addition, compared to urine or sputum samples which could be highly concentrated or diluted, cfDNA levels in plasma samples are considered to be more stable and therefore had potentially better stability in accuracy performance. Considering these advantages, we chose to focus on plasma, and aimed to better understand the accuracy performance of plasma sample in *KRAS* mutation detection in NSCLC, including potential impact of patient characteristics.

## 2 Materials and methods

### 2.1 Literature searching and selection of publication

Literature search was performed by BY and JZ in June 2022. Online literature databases (Pubmed, Embase, Cochrane Library, and Web of Science) were searched using keywords: “KRAS”, “plasma”, and “NSCLC”. Alternative spelling or abbreviations were also included in the literature search, e.g., non-small-cell lung cancer, non-small-cell lung carcinoma, NSCLCs, NSCLC’s, plasmas, and plasma’s (please see detailed searching strategy in **Supplementary Material**). Searching results were exported from each database. Duplicated literatures were then identified by matching titles, names of first author, or identification numbers (e.g., Pubmed ID) of literatures from different databases. After removing the duplicated literatures, the abstracts of the searching results were firstly screened to exclude irrelevant literatures. The full texts of the rest literatures were then downloaded and screened for eligible studies. The criteria used for the two screening steps were as follows. Inclusion criteria: all original studies testing *KRAS* mutation in paired plasma and tumor tissue samples of NSCLC. Exclusion criteria: 1) not a human study; 2) missing plasma or tumor tissue samples; 3) plasma and tumor tissue samples were not paired; 4) not testing *KRAS* mutation in either plasma or tissue samples; 5) lacking *KRAS* wild-type or *KRAS* mutated samples; 6) not an original study; 7) un-interpretable data; 8) not NSCLC samples. Accuracy data were then extracted from the *KRAS* mutation testing results of paired plasma and tumor tissue samples in the eligible studies, including numbers of true positive, false positive, false negative, and true negative. In addition, characteristics of patients or techniques were also extracted, including region and population of studies, tumor stage, and techniques used to test *KRAS* mutation in plasma and in tissue samples. All the eligible studies were evaluated by quality assessment of diagnostic accuracy studies 2 (QUADAS-2) (13). Any disagreement between the two investigators (BY and JZ) were solved by a third investigator (PC). PRISMA 2009 Checklist is included in **Supplementary Material**.

### 2.2 Statistical analysis

Statistical analysis was performed using Meta-DiSc 1.4 (14) and STATA 12.0 (STATA Corp.). Sensitivity, specificity, positive likelihood ratio (PLR), negative likelihood ratio (NLR), diagnostic odds ratio (DOR), and area under curve (AUC) of summary receiver operating characteristic (SROC) curve were pooled from the accuracy data extracted from the eligible studies. During the pooling, random effects model was used when significant heterogeneity was observed (*I^2^* ≥ 50% and *P* < 0.05), and fixed effects model was used when no significant heterogeneity was observed (14). In case of significant heterogeneity, threshold analysis and meta-regression were performed to find its possible sources. Deek’s funnel plot asymmetry test was performed to find potential publication bias in the eligible studies. *P* < 0.05 was considered statistically significant.

## 3 Results

### 3.1 Search results

**Figure 1:**
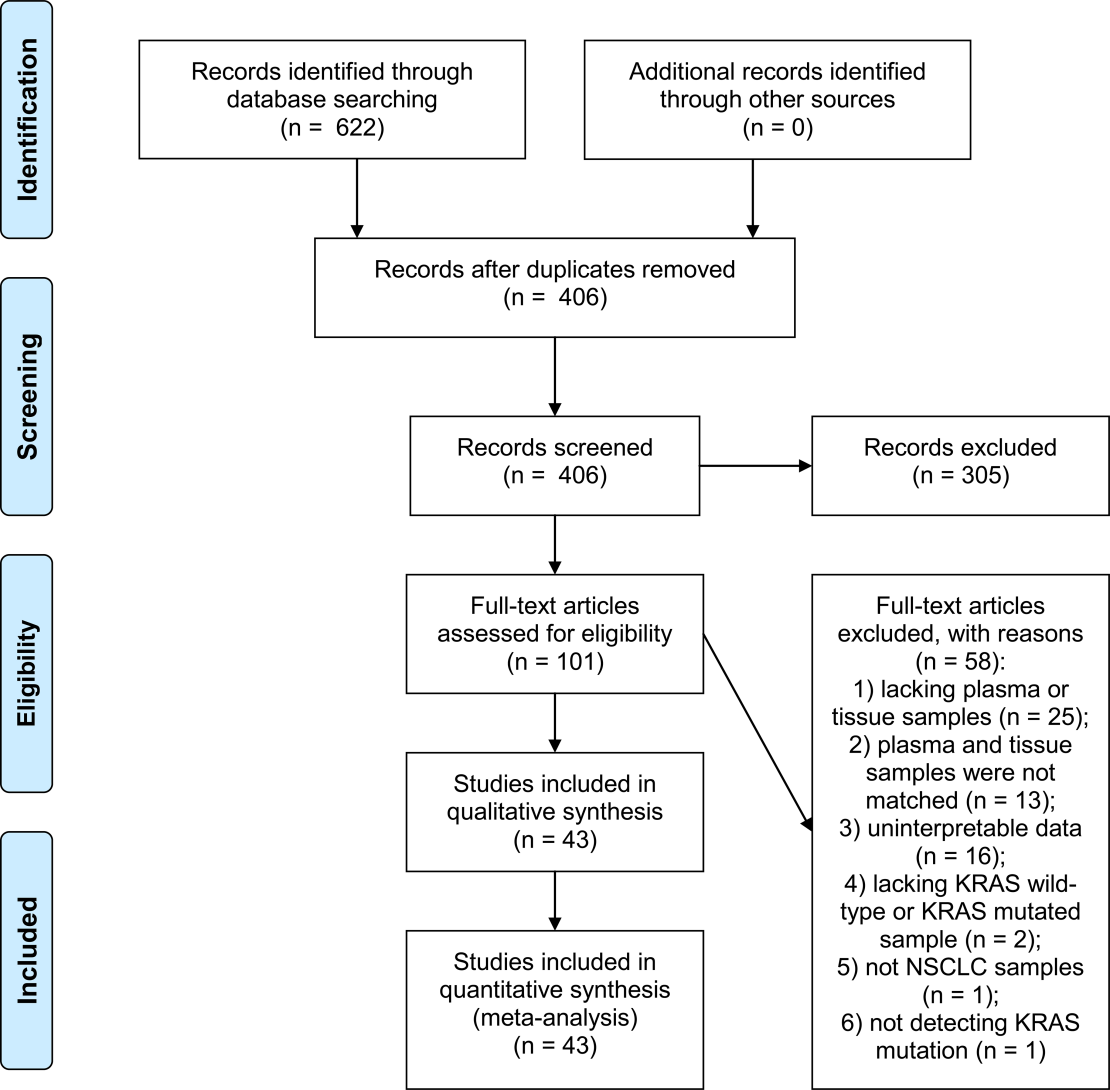
PRISMA 2009 flow diagram.

As shown in **Figure 1**, a total of 622 publications were identified after the literature search (Pubmed: 114; Embase: 333; Cochrane Library: 29; Web of Science: 146). After removing 216 duplicated literatures, titles and abstracts of the rest 406 publications were screened, and 305 irrelevant studies were excluded. Full text of the rest 101 publications were downloaded and carefully evaluated for their eligibility, and another 58 publications were further excluded. From the 43 eligible studies, accuracy data and other relevant information were extracted.

### 3.2 Review of eligible publications

Twenty-nine of the 43 eligible studies (**Table 1**) used next-generation sequencing (NGS) to detect *KRAS* mutation in plasma samples. In the rest 14 studies, 12 studies used PCR-based techniques, 1 study used pyrosequencing, and 1 study used MassARRAY.

**Table 1:** Summary of studies detecting *KRAS* mutation in paired plasma and tissue samples from NSCLC patients.

Author, year	Sample size	Detection method (plasma)	Detection method (tissue)	Region	Tumor stage	Race
Yin J et al., 2021 (15)	147	NGS (customized panel)	NGS (customized panel)	Asia	I-IV	Asian
Paweletz CP et al., 2016 (16)	48	NGS (customized panel)	not specified	America	III-IV	Caucasian
Narayan A et al., 2012 (17)	21	NGS (customized panel)	Sanger sequencing/clinical lab	America	I-IV	Caucasian
Couraud S et al., 2014 (18)	68	NGS (customized panel)	NGS (customized panel)	Europe	I-IV	Caucasian
Wang Z et al., 2017 (19)	103	NGS (cSMART)	ARMS-PCR	Asia	III-IV	Asian
Tran LS et al., 2019 (20)	40	NGS (Ultra-deep sequencing)	NGS (Ultra-deep sequencing)	Asia	III-IV	Asian
Yao Y et al., 2017 (21)	39	NGS (Agilent SureSelect)	NGS (Agilent SureSelect)	Asia	III-IV	Asian
Pritchett MA et al., 2019 (22)	147	NGS (Agilent SureSelect)	NGS (Agilent SureSelect)	America	III-IV	Caucasian
Liu L et al., 2018 (23)	65	NGS (customized panel)	NGS (customized panel)	Asia	III-IV	Asian
Li BT et al., 2019 (24)	110	NGS (customized panel)	NGS (customized panel)	America	IV	Caucasian
Chen Y et al., 2019 (25)	43	NGS (customized panel)	NGS (customized panel)	Asia	I-IV	Asian
Lin X et al., 2019 (26)	21	NGS (customized panel)	NGS (customized panel)	Asia	III-IV	Asian
Chen KZ et al., 2016 (27)	58	NGS (AmpliSeq Cancer Panel)	NGS (AmpliSeq Cancer Panel)	Asia	I-II	Asian
Xu S et al., 2016 (28)	42	NGS (AmpliSeq Cancer Panel)	NGS (AmpliSeq Cancer Panel)	Asia	III-IV	Asian
Pécuchet N et al., 2016 (29)	107	NGS (AmpliSeq Colon and Lung Cancer Research Panel v2)	NGS (AmpliSeq Colon and Lung Cancer Research Panel v2)	Europe	III-IV	Caucasian
Pasquale R et al., 2020 (30)	107	NGS (Oncomine Lung cfDNA assay)	NGS (Oncomine Solid Tumor DNA)	Europe	not disclosed	Caucasian
Mehta A et al., 2021 (31)	21	NGS (Oncomine Lung Cell-Free Total Nucleic Acid Assay)	NGS (Tag sequencing)	Asia	III-IV	Asian
Papadopoulou E et al., 2019 (32)	36	NGS (Oncomine Lung Cell-Free Total Nucleic Acid Assay)	NGS (AmpliSeq Colon and Lung Cancer Research Panel v2)	Europe	not disclosed	Caucasian
Nicolazzo C et al., 2021 (33)	38	NGS (Oncomine Lung Cell-Free Total Nucleic Acid Assay)	NGS (AmpliSeq Colon and Lung Cancer Research Panel v2)	Europe	not disclosed	Caucasian
Ma Y et al., 2020 (34)	28	NGS (AmoyDx Essential NGS panel)	NGS (AmoyDx Essential NGS panel)	Asia	I-IV	Asian
Garcia J et al., 2018 (35)	20	NGS (56G Oncology Panel Kit, Swift Biosciences)	NGS (customized AmqliSeq panel)	Europe	not disclosed	Caucasian
Remon J et al., 2019 (36)	88	NGS (InVisionSeq Lung, NeoGenomics)	Sanger sequencing or allele-specific technique	Europe	III-IV	Caucasian
Bauml JM et al., 2022 (37)	189	NGS (Guardant360)	PCR (therascreen KRAS RGQ PCR Kit)	America	I-IV	Caucasian
Thompson JC et al., 2016 (38)	50	NGS (Guardant360)	NGS (Illumina TruSeq Amplicon - Cancer Panel, or Penn Precision Panel)	America	II-IV	Caucasian
Leighl NB et al., 2019 (39)	282	NGS (Guardant360)	Standard of care (NGS, PCR, FISH and/or IHC, Sanger sequencing	America	III-IV	Caucasian
Lam VK et al., 2021 (40)	76	NGS (Guardant360)	not specified	America	III-IV	Caucasian
Qvick A et al., 2021 (41)	52	NGS (AVENIO ctDNA Surveillance kit)	NGS (AmpliSeq Colon and Lung Cancer Research Panel v2, or AVENIO FFPE Surveillance kit (sufficient sample), or qPCR and FISH (insufficient samples)	Europe	I-IV	Caucasian
Jiao XD et al., 2021 (42)	185	NGS (LungPlasma panel)	NGS (OncoScreen Plus panel)	Asia	III-IV	Asian
Guo N et al., 2016 (43)	41	NGS (SV-CA50-ctDNA panel, San Valley Biotech Inc.)	NGS (SV-CA50-ctDNA panel, San Valley Biotech Inc.)	Asia	I-IV	Asian
Michaelidou K et al., 2020 (44)	96	ddPCR	Sanger sequencing	Europe	III-IV	Caucasian
Oxnard GR et al., 2014 (45)	31	ddPCR	Central lab	America	III-IV	Caucasian
Sacher AG et al., 2016 (46)	87	ddPCR	not specified	America	III-IV	Caucasian
Mellert H et al., 2017 (47)	100	ddPCR	not specified	America	III-IV	Caucasian
Cho MS et al., 2020 (48)	36	PCR-based multiplex assay (PANAmutyper)	PCR-based multiplex assay (PNAmutyper)	Asia	I-IV	Asian
Han JY et al., 2016 (49)	135	PCR-based multiplex assay (PANAmutyper)	PCR-based direct DNA sequencing	Asia	III-IV	Asian
Wang S et al., 2010 (50)	273	PCR-RFLP	Direct sequencing	Asia	I-IV	Asian
Gautschi O et al., 2007 (51)	9	PCR-RFLP	Sanger sequencing	Europe	I-IV	Caucasian
Zhang H et al., 2013 (52)	86	Multiplex PCR (SurPlex MEL, SurExam Biotech, Inc)	Multiplex PCR (SurPlex-xTAG70plex, SurExam Biotech, Inc)	Asia	III-IV	Asian
Punnoose EA et al., 2012 (53)	18	Multiplex PCR (customized primers) + TaqMan assay or DxS kit	not specified	USA & Australia	not disclosed	Caucasian
Mack PC et al., 2009 (54)	49	ARMS	ARMS	America	III-IV	Caucasian
Campos CDM et al., 2018 (55)	3	solid phase extraction + PCR/LDR	PCR/LDR	America	III-IV	Caucasian
Kulasinghe A et al., 2021 (56)	103	MassARRAY (UltraSEEK lung panel, Agena Biosciences)	not specified	Australia	I-IV	Caucasian
Li XQ et al., 2014 (57)	43	pyrosequencing	pyrosequencing	Asia	III-IV	Asian

NGS, next generation sequencing; PCR, polymerase chain reaction; PCR-RFLP, PCR-restriction fragment length polymorphism; ddPCR, digital droplet PCR; ARMS, Amplification Refractory Mutation System.

#### 3.2.1 NGS

In the eligible studies using NGS, sensitivities ranged from 25% to 100%, and specificities and concordance rates were relatively higher, ranging from 64% to 100% and from 52.63% to 100%, respectively.

Twelve studies used customized NGS panels, in which 5 studies used amplicon-based targeted sequencing (15–19). In the study by Yin (15), *KRAS* mutation detected in tumor tissue samples were all detected in paired plasma samples, resulting in 100% sensitivity. The specificity and concordance rate were 99.24% and 99.32%, respectively. Similarly, study by Narayan (17) showed perfect matching (100% concordance rate) of *KRAS* mutation results between plasma and tissue samples. However, study by Paweletz (16) and by Couraud (18) showed much lower sensitivity (54.55% and 75%, respectively), although high specificity (100%) was observed. In the study by Wang Z (19), circulating single-molecule amplification and resequencing technology (cSMART) showed sensitivity of 58.82%, specificity of 100%, and concordance rate of 93.20%. The large variations in the sensitivity of *KRAS* mutation detection in plasma samples may be due to the small number of patients included in these studies.

The rest 7 studies used hybridization-based targeted sequencing (20–26). A customized panel from xGen (Integrated DNA Technologies) showed perfect match between plasma and tumor tissue results (100% concordance rate) (20). Studies by Yao (21) and Pritchett (22) used a hybridization-based target enrichment method from Agilent Technologies (SureSelect). The two studies showed similar concordance rates (91.16% and 97.44%). Studies by Liu (23), Li BT (24), Chen Y (25), and Lin (26) also used hybridization-based capture methods to enrich customized gene panels for NGS sequencing of plasma samples. The concordance rates of those studies were all high, ranging from 93.02% to 96.92%.

Besides customized NGS panels, several commercial NGS panels were also used, such as AmpliSeq panels, Oncomine panels, AmoyDx Essential NGS panel, 56G Oncology Panel, InVisionSeq Lung, Guardant360, AVENIO ctDNA Surveillance kit, LungPlasma panel, and SV-CA50-ctDNA panel. AmpliSeq Cancer Panel (Thermo Fisher Scientific) was used in two studies (27, 28). However, the results varied greatly between them. Sensitivity, specificity, and concordance rate were 60%, 96.23%, and 93.10% in Chen KZ’s study (27), and 100%, 83.33%, and 85.71% in Xu’s study (28). AmpliSeq Colon and Lung Cancer Research Panel v2 showed sensitivity of 62.96%, specificity of 100%, and concordance rate of 90.65% (29). Oncomine Lung cfDNA Assay (Thermo Fisher Scientific) showed sensitivity, specificity, and concordance rate of 61.54%, 93.83%, and 85.98%, respectively (30). Oncomine Lung Cell-Free Total Nucleic Acid Assay (Thermo Fisher Scientific) was used in three studies, and accuracy results varied greatly: sensitivity from 30.77% to 81.82%, specificity from 64% and 100%, and concordance rate from 52.63% to 94.44% (31–33). AmoyDx Essential NGS panel (Amoy Diagnostics) was used in a 28-patient cohort, and the sensitivity, specificity, and concordance rate were 66.67%, 96%, and 92.86%, respectively (34). Studies by Garcia (35) and Remon (36) also used amplicon-based targeted sequencing techniques, including 56G Oncology Panel (Swift Biosciences), InVisionSeq Lung (NeoGenomics), respectively. Results showed sensitivity of 64.29% and 88%, specificity of 83.33% and 88.89%, and concordance rate of 70% and 88.64%.

Four studies validated the accuracy of Guardant360 in detecting *KRAS* mutation in plasma samples (37–40). Sensitivity ranged from 66.67% to 87.50%. Specificity ranged from of 74.81% to 100%, and concordance rate ranged from 75.89% to 98%. AVENIO ctDNA Surveillance kit (Roche) is also a commercial panel using hybridization-based target enrichment. A study using AVENIO ctDNA Surveillance kit showed sensitivity of 72.73%, specificity of 100%, and concordance rate of 94.23% (41).

In the rest two studies using commercial NGS panels, detailed target enrichment method was not disclosed. Studies by Jiao (42) used LungPlasma NGS panel (Burning Rock Biotech), and sensitivity, specificity, and concordance rate were 68.97%, 99.36%, and 94.59%. Guo (43) used SV-CA50-ctDNA panel (San Valley Biotech), and results showed 50% sensitivity, 97.44% specificity, and 95.12% concordance rate.

#### 3.2.2 PCR-based techniques

A total of 4 studies used digital droplet PCR (ddPCR) to detect *KRAS* mutation in plasma samples (44–47). Although ddPCR is a sensitive technique which could detect genetic mutations as low as 0.01%, the results of these studies did not show high accuracy of ddPCR in plasma-based *KRAS* mutation detection. Sensitivity ranged from 51.43% to 87.88%, and specificity ranged from 88.52% to 100%, resulting in concordance rates from 75% to 96%.

Other than ddPCR, several PCR-based techniques were also used to detect *KRAS* mutation in plasma samples, such as PANAmutyper, PCR-restriction fragment length polymorphism (PCR-RFLP), multiplex PCR, Amplification Refractory Mutation System (ARMS), and PCR/ligase detection reaction (LDR) technique. Overall, those PCR-based techniques were mostly used in early studies, which showed sensitivity ranging from 33.33% to 100%, specificity from 50% to 100%, and concordance rate from 55.56% to 100%.

PANAmutyper is a multiplex PCR method which increases sensitivity through suppressing amplification of wild-type DNA using specific peptide nucleic acids (PNA) (48). In the two studies using PANAmutyper, the sensitivity was 33.33% and 50%, and specificity was 100% and 89.43%, resulting in concordance rates of 88.89% and 85.93%, respectively (48, 49).

In the two studies using PCR-RFLP, accuracy results varied greatly. In Wang S’s study (50), the sensitivity, specificity, and concordance rate were 76.67%, 95.06%, and 93.04%, respectively. In the study of Gautschi (51), these numbers were 50%, 66.67%, and 55.56%, respectively.

Multiplex PCR was used in two studies. Study by Zhang (52) used SurExam MEL (SurExam Biotech), a typical commercial multiplex PCR, to detect *KRAS* mutation in plasma samples, and sensitivity, specificity, and concordance rate were 33.33%, 98.80%, and 96.51%. In the study by Punnoose (53), the *KRAS* mutation results of plasma samples matched perfectly with tissue samples (100% concordance rate).

An early study by Mack (54) used KRAS Scorpion-ARMS test kit (DxS Ltd), and results showed 50% sensitivity, 100% specificity, and 97.96% concordance rate.

Campos (55) and colleagues developed a microfluidic solid-phase extraction device to extract cfDNA, which were then analyzed using PCR/LDR technique. Only 3 NSCLC samples were tested in the study, and the results showed 100% sensitivity, 50% specificity, and 66.67% concordance rate.

#### 3.2.3 MassARRAY and pyrosequencing

UltraSEEK lung panel (Agena Biosciences), a commercial MassARRAY panel, was used in a 103-patient cohort, and sensitivity, specificity, and concordance rate were 62.96%, 92.11%, and 84.47%, respectively (56). Pyrosequencing was used in an early study (57), and sensitivity and specificity were 75% and 100%, respectively, resulting in a concordance rate of 97.67%.

In all, the 43 eligible studies compared *KRAS* mutation status in paired plasma and tissue samples from 3341 NSCLC patients. Thirty-nine of the 43 eligible studies (39/43) showed high specificity (≥ 80%), and 37 studies showed high concordance rate (≥ 80%). However, high sensitivity (≥ 80%) was only observed in 14 out of 43 studies.

### 3.3 Quality assessment of eligible studies

Quality assessment of eligible studies was performed using QUADAS-2. As shown in **Table 2**, the 43 eligible studies showed overall good quality, with high risk observed in only 2 studies (both in flow and timing). In the assessment of risk of bias, percentage of low risk ranged from 46.51% (n = 20, Index test) to 69.77% (n = 30, both patient selection and reference standard). In the application concerns, no high risk was observed, and percentage of low risk ranged from 83.72% (n = 36, reference standard) to 86.05% (n = 37, both patient selection and index test).

**Table 2:** QUADAS-2 assessment of eligible studies.

Author, year	Risk of bias					Applicability concerns		
	Patient selection	Index test	Reference standard	Flow and timing		Patient selection	Index test	Reference standard
Yin J et al., 2021 (15)	low	unclear	low	low		low	low	low
Paweletz CP et al., 2016 (16)	low	low	low	low		low	low	low
Narayan A et al., 2012 (17)	low	unclear	low	unclear		unclear	low	low
Couraud S et al., 2014 (18)	low	unclear	unclear	unclear		low	low	unclear
Wang Z et al., 2017 (19)	low	low	low	unclear		low	unclear	unclear
Tran LS et al., 2019 (20)	low	unclear	unclear	low		low	low	low
Yao Y et al., 2017 (21)	unclear	unclear	low	low		low	low	low
Pritchett MA et al., 2019 (22)	low	unclear	low	unclear		low	low	low
Liu L et al., 2018 (23)	low	unclear	unclear	low		low	low	low
Li BT et al., 2019 (24)	low	low	low	unclear		low	low	low
Chen Y et al., 2019 (25)	low	unclear	low	unclear		unclear	low	low
Lin X et al., 2019 (26)	unclear	low	low	unclear		low	low	low
Chen KZ et al., 2016 (27)	unclear	unclear	low	low		low	unclear	low
Xu S et al., 2016 (28)	low	low	low	low		low	low	low
Pécuchet N et al., 2016 (29)	low	low	low	high		low	unclear	unclear
Pasquale R et al., 2020 (30)	low	low	low	low		low	low	low
Mehta A et al., 2021 (31)	unclear	unclear	low	unclear		low	low	low
Papadopoulou E et al., 2019 (32)	low	unclear	low	unclear		low	low	low
Nicolazzo C et al., 2021 (33)	unclear	unclear	low	unclear		low	low	low
Ma Y et al., 2020 (34)	unclear	low	unclear	unclear		low	low	low
Garcia J et al., 2018 (35)	low	unclear	low	low		low	unclear	unclear
Remon J et al., 2019 (36)	low	low	unclear	unclear		unclear	low	low
Bauml JM et al., 2022 (37)	low	unclear	low	low		low	low	low
Thompson JC et al., 2016 (38)	low	low	low	low		low	low	unclear
Leighl NB et al., 2019 (39)	low	low	low	unclear		low	unclear	unclear
Lam VK et al., 2021 (40)	unclear	unclear	unclear	unclear		unclear	low	low
Qvick A et al., 2021 (41)	unclear	low	unclear	unclear		low	low	low
Jiao XD et al., 2021 (42)	low	unclear	low	low		low	unclear	low
Guo N et al., 2016 (43)	low	low	low	low		low	low	low
Michaelidou K et al., 2020 (44)	low	unclear	low	low		low	low	low
Oxnard GR et al., 2014 (45)	unclear	unclear	unclear	unclear		low	low	low
Sacher AG et al., 2016 (46)	low	low	low	low		low	low	low
Mellert H et al., 2017 (47)	unclear	unclear	unclear	unclear		low	low	low
Cho MS et al., 2020 (48)	low	low	low	unclear		low	low	low
Han JY et al., 2016 (49)	low	unclear	low	low		low	low	unclear
Wang S et al., 2010 (50)	low	low	low	low		low	low	low
Gautschi O et al., 2007 (51)	low	low	unclear	unclear		low	low	low
Zhang H et al., 2013 (52)	low	low	low	low		low	low	low
Punnoose EA et al., 2012 (53)	unclear	unclear	unclear	high		unclear	low	low
Mack PC et al., 2009 (54)	unclear	low	unclear	low		low	low	low
Campos CDM et al., 2018 (55)	unclear	unclear	low	unclear		unclear	low	low
Kulasinghe A et al., 2021 (56)	low	unclear	low	low		low	low	low
Li XQ et al., 2014 (57)	low	low	unclear	low		low	low	low

low, low risk; unclear, unclear risk; high, high risk.

### 3.4 Meta-analysis

**Figure 2:**
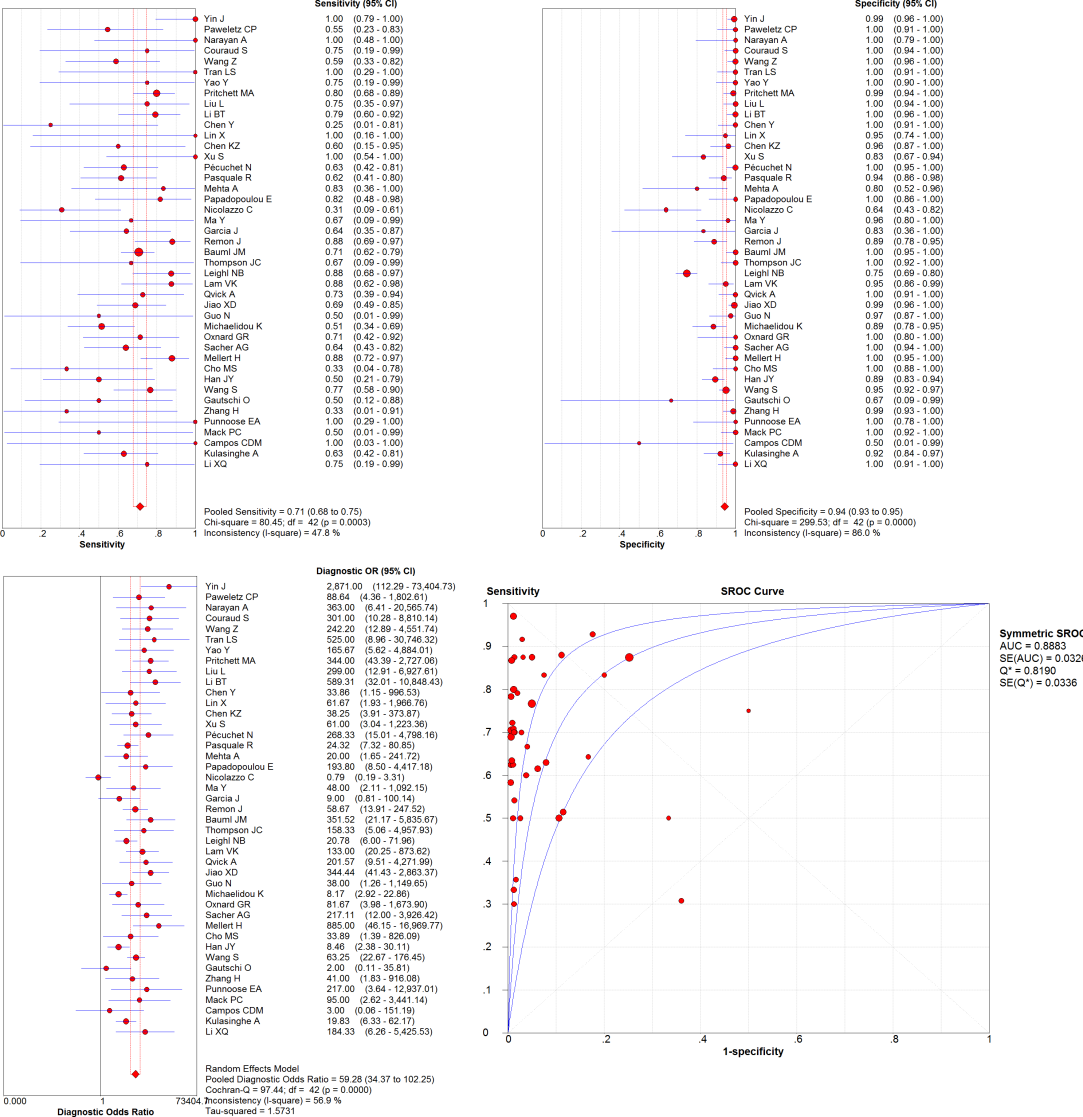
Pooled sensitivity, specificity, DOR, and SROC curve of eligible studies.

From the 43 eligible studies, we pooled the *KRAS* mutation detection results from paired plasma and tissue samples of 3341 patients with NSCLC. The overall sensitivity and specificity were 0.71 [95% confidence interval (CI): 0.68-0.75] and 0.94 (95%CI: 0.93-0.95), respectively. The pooled DOR was 59.28 (95%CI: 34.37-102.25), and AUC of SROC curve was 0.8883. Please see **Table 3** and **Figure 2** for details.

**Table 3:** Meta-analysis results.

	No. of studies	Sensitivity	Specificity	PLR	NLR	DOR	AUC of SROC curve
**Overall**	43	0.71(0.68-0.75)	0.94(0.93-0.95)	16.27(10.08-26.25)	0.36(0.30-0.43)	59.28(34.37-102.25)	0.8883
Techniques used for plasma sample							
**NGS**	29	0.73(0.69-0.77)	0.94(0.93-0.95)	20.99(10.68-41.23)	0.33(0.26-0.41)	82.60(40.62-167.96)	0.9162
**PCR-based techniques**	12	0.66(0.59-0.74)	0.95(0.94-0.97)	9.88(4.60-21.19)	0.42(0.31-0.58)	31.58(11.88-83.95)	0.7888
**ddPCR**	4	0.68(0.59-0.77)	0.97(0.93-0.99)	26.46(2.68-261.05)	0.33(0.18-0.59)	85.60(6.80-1078.05)	0.2741
**Other PCR-based techniques**	8	0.63(0.50-0.75)	0.95(0.93-0.97)	7.61(3.16-18.31)	0.40(0.29-0.55)	22.01(11.18-43.33)	0.8147
Region							
**Asia**	18	0.71(0.63-0.78)	0.97(0.95-0.98)	18.00(9.96-32.53)	0.32(0.25-0.40)	63.84(38.95-104.65)	0.9381
**America**	13	0.76(0.71-0.81)	0.92(0.90-0.94)	31.28(5.36-182.47)	0.25(0.20-0.30)	111.35(56.05-221.20)	0.9272
**Europe**	10	0.63(0.56-0.71)	0.93(0.91-0.95)	7.42(3.17-17.41)	0.43(0.29-0.62)	22.62(6.69-76.49)	0.7013
Tumor stage							
**I-IV**	13	0.71(0.65-0.77)	0.97(0.96-0.98)	22.11(13.39-36.52)	0.39(0.28-0.54)	64.59(34.43-121.17)	0.9273
**III-IV**	24	0.73(0.69-0.78)	0.93(0.92-0.94)	18.68(9.26-37.69)	0.29(0.25-0.34)	54.70(36.59-81.75)	0.9086
Race of patients							
**Asian**	18	0.71(0.63-0.78)	0.97(0.95-0.98)	18.00(9.96-32.53)	0.32(0.25-0.40)	63.84(38.95-104.65)	0.9381
**Caucasian**	25	0.72(0.68-0.75)	0.92(0.91-0.94)	14.85(7.39-29.84)	0.34(0.27-0.42)	53.73(24.95-115.69)	0.8445

PLR, positive likelihood ratio; NLR, negative likelihood ratio; DOR, diagnostic odds ratio; AUC, area under curve; SROC, summary receiver operating characteristic; PCR, polymerase chain reaction; NGS, next generation sequencing; ddPCR, digital droplet PCR.

Since significant heterogeneity (*I^2^* ≥ 50% and *P* < 0.05) was observed, we further analyzed its possible sources. Analysis of diagnostic threshold showed no significant threshold effect (spearman correlation coefficient = 0.058, *P* = 0.714). Meta-regression revealed that inter-study heterogeneity was associated with techniques used for plasma sample (*P* = 0.0388), but not with techniques used for tissue sample (*P* = 0.1280), region of study (*P* = 0.3299), tumor stage (*P* = 0.3049), or race of patients (*P* = 0.7798).

Subgroup analysis was then performed on different techniques used for plasma sample. The 43 eligible studies were grouped into three subgroups: NGS, PCR-based techniques, and other techniques. Meta-analysis was performed in each subgroup except other techniques due to limited number (only two) of studies in that subgroup. As shown in **Table 3**, compared to PCR-based techniques, NGS showed overall better accuracy: sensitivity of 0.73 (95%CI: 0.69-0.77), specificity of 0.94 (95%CI: 0.93-0.95), DOR of 82.60 (95%CI: 40.62-167.96), and AUC of SROC curve of 0.9162. After further dividing the group of PCR-based techniques into two subgroups (ddPCR and other PCR-based techniques), ddPCR showed higher sensitivity [0.68 (95%CI: 0.59-0.77)], specificity [0.97 (95%CI: 0.93-0.99)], and DOR [85.60 (95%CI: 6.80-1978.05)], but much lower AUC of SROC curve (0.2741).

Subgroup analysis was also performed on the region of studies, including Asia, America, Australia, and Europe. Australia was excluded from the subgroup analysis due to limited number of studies in the subgroup. In the other three subgroups, studies performed in America showed overall best accuracy, with pooled sensitivity of 0.76 (95%CI: 0.71-0.81), specificity of 0.92 (95%CI: 0.90-0.94), DOR of 111.35 (95%CI: 56.05-221.20), and AUC of SROC curve of 0.9272.

Twenty-four of the 43 eligible studies used late-stage (stage III and IV) NSCLC samples, and 13 studies used NSCLC samples of any stage (stage I to IV). As shown in **Table 3**, pooled accuracy results of the two subgroups (stage III-IV *versus* stage I-IV) did not differ much from each other. However, this result should be treated carefully because although early-stage NSCLC samples were involved, majority of the samples were still late-stage in stage I-IV subgroup. The rest 6 studies were not involved in the subgroup analysis, including 1 study using early-stage (I and II) NSCLS samples only, and 5 studies which did not disclose the tumor stage of samples.

Majority of the 43 eligible studies were conducted using samples from Caucasian patients, and the rest studies used samples of Asian patients. Between the two subgroups, pooled accuracy data were similar (see **Table 3**).

**Figure 3:**
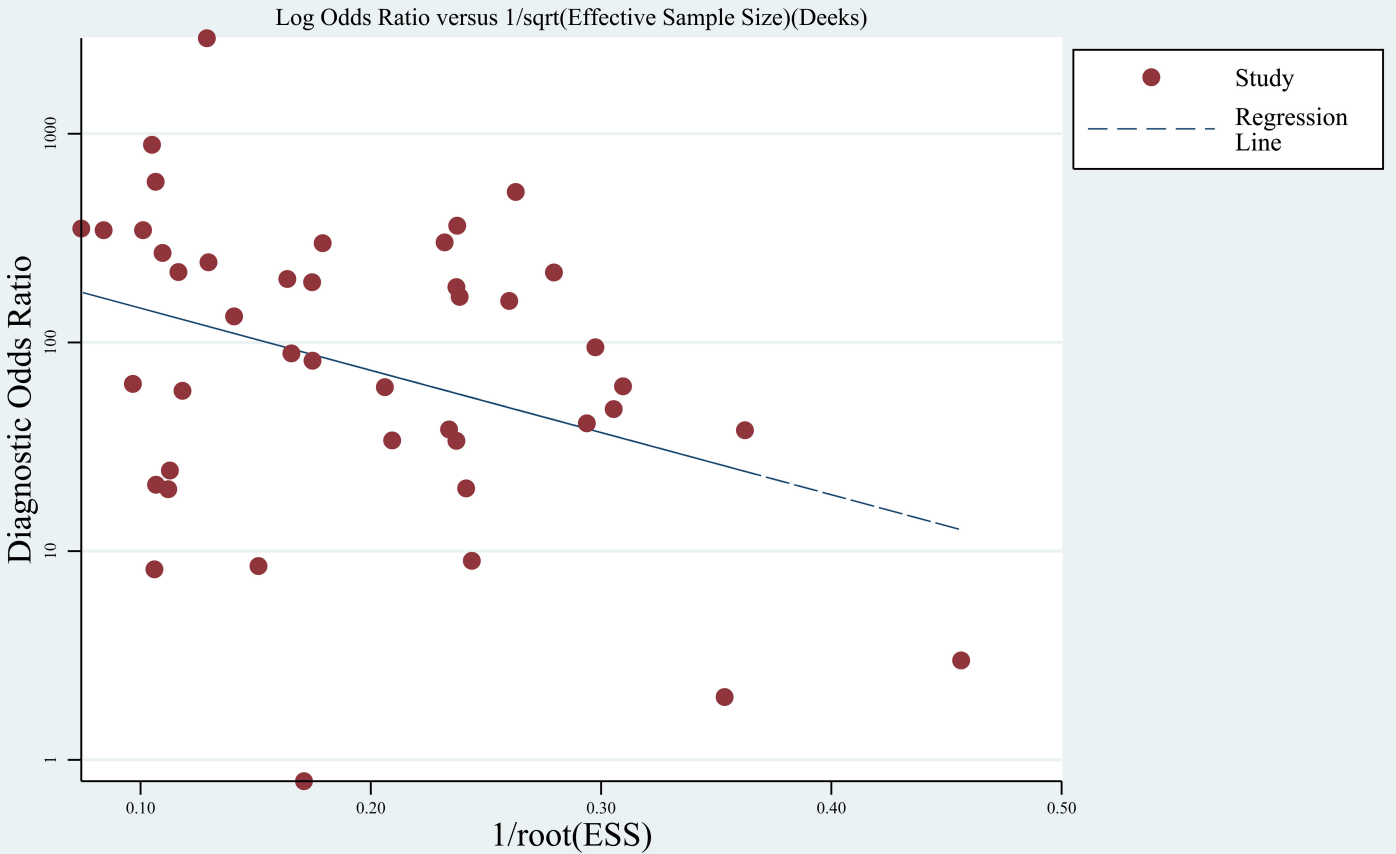
Deek’s funnel plot.

Publication bias was evaluated using Deek’s funnel plot (**Figure 3**). The results indicated no significant publication bias (*P* = 0.097).

## 4 Discussion

Before anti-EGFR therapies are given to NSCLC patients, it is important to determine whether the tumor carries *KRAS* mutation since it may lead to resistance to anti-EGFR therapies. Moreover, determination of *KRAS* mutation status is also required before the usage of KRAS (G12C) inhibitor, e.g., Sotorasib. Tumor tissue samples are the “gold standard” in the determination of *KRAS* mutation. However, tumor tissue samples are sometimes not available, and cfDNA-containing samples (e.g., plasma, urine, saliva, etc.) have been intensively investigated as surrogates for tissue samples. A recently-published systemic review and meta-analysis by Palmieri summarized the performance of cfDNA-containing samples in detecting *KRAS* mutation in NSCLC (12). Due to the higher and more stable levels of cfDNA in plasma compared to other cfDNA-containing sample types, we focused solely on plasma in this systemic review and meta-analysis, and investigated its accuracy in determining tumor *KRAS* mutation status in NSCLC.

In order to investigate the accuracy of *KRAS* mutation detection using plasma samples, several previous studies compared *KRAS* mutation results in paired plasma and tissue samples from patients with NSCLC. After database searching and screening, we identified 43 eligible studies. After pooling the *KRAS* mutation status from 3341 patients with NSCLC, the results showed overall moderate sensitivity (0.71) and high specificity (0.94). Other important indicators of diagnostic accuracy, DOR and AUC of SROC curve, were also high (59.28 and 0.8883, respectively). Although with moderate sensitivity, these results indicated overall high accuracy of plasma samples in detecting *KRAS* mutation. In the systemic review and meta-analysis by Palmieri (12), the pooled sensitivity and specificity were 0.71 and 0.93, respectively, and DOR was 35.24, which were similar to the findings of our study.

Since significant inter-study heterogeneity was observed during the pooling (*I^2^* ≥ 50% and *P* < 0.05), we investigated its possible sources. Analysis of diagnostic threshold did not indicate significant threshold effect. Meta-regression revealed significant association between inter-study heterogeneity and techniques used for plasma sample. This is different from Palmieri’s study, in which detection method did not contribute to heterogeneity (12). No significant association was shown between heterogeneity and other covariates (techniques used for tissue sample, region of study, tumor stage, and race of patients).

Different from Palmieri’s study, we further conducted subgroup analysis. Subgroup analysis on technique used for plasma sample was firstly performed. After pooling the accuracy results, we found that NGS outperformed PCR-based techniques in many accuracy parameters, including sensitivity (0.73), DOR (82.60), and AUC of SROC curve (0.9162). We further divided the group of PCR-based techniques into two groups: ddPCR and other PCR-based techniques. Compared to NGS, ddPCR showed similar sensitivity (0.68), specificity (0.97), and DOR (85.60), except for surprisingly low AUC of SROC curve (0.2741) which was possibly due to the limited number of studies in this subgroup (**Table 3**).

We also performed subgroup analysis on region of study. Studies performed in Asia showed the highest AUC of SROC curve (0.9381). Studies performed in America showed the highest sensitivity (0.76) and DOR (111.35), and similar AUC of SROC curve with Asia (0.9272), indicating overall the highest accuracy of the studies from America.

Late-stage tumors was reported to be associated with significantly higher fraction of circulating tumor DNA (ctDNA) in cfDNA (58), which may indicate potentially better performance of genetic testing using these samples. In the 43 eligible studies, involvement of early-stage samples did not significantly influence the accuracy results. However, this result should be treated with care because numbers of early-stage samples were much smaller than late-stage samples in a large proportion of these studies. Race of patients also did not show significant impact on the accuracy results. The performance of *KRAS* mutation testing using plasma was similar between Asian and Caucasian patients. Significant publication bias was not observed using Deek’s funnel plot asymmetry test.

In summary, results of this systemic review and meta-analysis indicated overall high accuracy of plasma samples in predicting *KRAS* mutation results of paired NSCLC tumor tissue samples. Plasma could serve as surrogates when tissue samples are not available, although it may miss a small proportion of patients carrying *KRAS* mutation considering its moderate sensitivity. Among different techniques, NGS showed the best accuracy. Although majority of accuracy results were comparable to NGS, ddPCR suffered from its low AUC of SROC curve. Therefore, NGS is recommended in the detection of *KRAS* mutations in plasma samples of patients with NSCLC, especially when multiple genetic variations are tested considering the high-throughput of the technology. Limitation of this study may be the small number of studies in the ddPCR subgroup and limited numbers of early-stage tumor samples used in some studies, which must be treated carefully. In addition, although different techniques are generally thought to have similar performance in tumor samples considering the high abundance of DNA, it may still cause potential bias. Large prospective studies are required to further validate the results of this study.

## Data availability statement

The original contributions presented in the study are included in the article/**Supplementary Material**. Further inquiries can be directed to the corresponding author.

## Author contributions

PC, BY, and DY contributed to conception and design of the study. BY and JZ organized the database. PY performed the statistical analysis. PC wrote the first draft of the manuscript. BY, JZ, and PY wrote sections of the manuscript. All authors contributed to manuscript revision, read, and approved the submitted version.
