# Supplementary material for: Detection of KRAS mutation using plasma samples in non-small-cell lung cancer: a systematic review and meta-analysis
Source: Front Oncol. 2023 Jul 6;13:1207892. doi: 10.3389/fonc.2023.1207892 (PMC10357383; doi:10.3389/fonc.2023.1207892)
Supplement: Supplementary file 3 [file Table_3.docx]

| **Supplementary Table 3** Search strategy | |
| --- | --- |
| Database | Search strategy |
| Pubmed | "KRAS"[All Fields] AND ("plasma"[MeSH Terms] OR "plasma"[All Fields] OR "plasmas"[All Fields] OR "plasma’s"[All Fields]) AND ("carcinoma, non small cell lung"[MeSH Terms] OR ("carcinoma"[All Fields] AND "non small cell"[All Fields] AND "lung"[All Fields]) OR "non-small-cell lung carcinoma"[All Fields] OR "nsclc"[All Fields] OR "nsclc’s"[All Fields] OR "nsclcs"[All Fields]) |
| Embase | ('kras'/exp OR kras) AND ('plasma'/exp OR plasma) AND ('non-small-cell lung cancer'/exp OR 'non-small-cell lung cancer') AND 'article'/it |
| Cochrane Library | (plasma):ti,ab,kw AND (KRAS):ti,ab,kw AND (non-small-cell lung cancer):ti,ab,kw |
| Web of Science | ((ALL=(KRAS)) AND ALL=(plasma)) AND ALL=(non-small-cell lung cancer) |
